# A Simple and Fast Procedure to Determine 3-Nitropropanoic Acid and 3-Nitropropanol in Freeze Dried Canadian Milkvetch (*Astragalus canadensis*)

**DOI:** 10.3390/toxins9070204

**Published:** 2017-06-29

**Authors:** Huaizhi Liu, Suqin Shao, Mike Schellenberg

**Affiliations:** 1 Guelph Research and Development Centre, Agriculture and Agri-Food Canada, Guelph, ON N1G 5C9, Canada; huaizhi.liu@agr.gc.ca; 2 Swift Current Research and Development Centre, Agriculture and Agri-Food Canada, Swift Current, SK S9H 3X2, Canada; mike.schellenberg@agr.gc.ca

**Keywords:** Canadian milkvetch, 3-nitropropanoic acid, 3-nitropropanol, plant toxin

## Abstract

Canadian milkvetch (*Astragalus canadensis*) is a North American plant species in the legume family and some of this plant is fatally poisonous to livestock. The poisoning is attributed to the natural occurrence of notrotoxins, i.e., 3-nitropropanoic acid and 3-nitropropanol, present as aglycones and conjugated forms in the plant. Those compounds cause nitrite oxidization of hemoglobin and inhibition of cellular metabolism. To determine the toxicity of the plant, it is very important to develop an analytical method for the contents of the compounds in the plant. In this study, we have successfully developed an extraction procedure followed by HPLC-UV analysis to simultaneously analyze notrotoxins. The aglycones could be released from its conjugated forms in the freeze dried plant and extracted by water at room temperature. An HPLC-UV method using a Phenomenex Kinetex 2.6 μ F5 100 Å 100 × 4.6 mm column with pH 3.5 phosphonate buffer as mobile phase have been developed and validated for the detection of the two compounds at 210 nm. This developed procedure for the analysis of 3-nitropropanoic acid and 3-nitropropanol has proven simple and efficient and it has been successfully applied for batch sample analysis.

## 1. Introduction

Canadian milkvetch (*Astragalus canadensis*) is a native North American plant species in the legume family and can be found throughout many parts of Canada and the United States. Although this plant could be potentially used as forage, Canadian milkvetch could be fatally poisonous to livestock [1]. The poisoning is attributed to the natural occurrence of toxic aliphatic nitro compounds, glycosides of 3-nitropropanol (3-NPOH) and glucose esters of 3-nitropropanoic acid (3-NPA) [2], including miserotoxin, cibarian, karakin, hiptagin, and free 3-NPOH and 3-NPA. Additionally, *Astragalus canadensis* also contains oxotetrahydrofuranyl and isoxazolinone esters of 3-NPA [3]. Upon hydrolysis, miserotoxin will release 3-NPOH, Cibarian, Karakin, and hiptagin and other esters of 3-NPA results in the formation of 3-NPA. Both 3-NPOH and 3-NPA have been found toxic to chicks, sheep, horses, and cattle [4].

Although the toxic property of *A. canadensis* has been well known, limited research has been conducted on this plant. Pioneer work done in the 70s and early 80s focused on the identification of those toxic compounds and limited work on the quantification of those compounds was done through spectrophotometric method [2,5,6]. For example, miserotoxin, 3-nitro-1-propyl β-d-glucopyranoside was firstly identified in *Astragalus miser* by Stermitz and Lowry (1972) [7] and later semi-quantified in *A. canadenis* by Williams at al. (1975) using TLC method [2]. Spectrophotometric method, which was used for quantification, was based on the nitrite ion released from nitro compounds with sulfanilic acid and 1-naphthylamine hydrochloride. However, the method was not specific and could interact with the pigments co-extracted from plants, and the reaction with 3-NPA and 3-NPOH was also found to be incomplete [8]. Ever since the mid-80s, not much work has occurred on the analysis of those compounds in the plant although some work was found on the analysis of 3-NPA and 3-NPOH in other matrices. For examples, previously, gas chromatography (GC) had been used for the analysis of 3-NPA in cheese after liquid-liquid extraction and derivatization with pentafluorobenzyl [9]. High performance liquid chromatography (HPLC) was used to quantify 3-NPA and 3-NPOH in bovine plasma after complicated sample preparation, including deproteinization, liquid-liquid extraction, and solid phase extraction [10].

Since the toxicity of *A. canadensis* varies a lot between different genomic backgrounds and/or growing conditions and there is increasing interest in using *A. canadensis* as forage, it is essential to develop a reliable analytical method to determine the contents of 3-NPA and 3-NPOH in the plant. Both 3-NPA and 3-NPOH are soluble in a variety of polar and non-polar solvents, including water, ethanol and diethyl ether. However, considering the complexity of the form of both compounds in the plant material, and the complexity of the material itself, the optimal solvent that can be used for efficient extraction of the compounds in the sample needs to be identified through systematic study. Additionally, due to the high solubility of both compounds in water, they are hard to be retained and separated in commonly used reverse phase C18 HPLC column. An HPLC method needs to be developed for the analysis of the two compounds.

## 2. Results and Discussion

### 2.1. Extraction of 3-NPA and 3-NPOH from Canadian Milkvetch

Pure organic solvent ethanol, acetone or acetonitrile was found not able to extract neither 3-NPA or 3-NPOH from Canadian milkvetch samples when 10 mL solvent was used for 0.50 g freeze dried samples, even for a long time (48 h). However, when water or HCl was mixed with organic solvent, some 3-NPA was detected in the extract, indicating the role of water or HCl in extraction of the two compounds from the sample. Since pure 3-NPA or 3-NPOH are soluble in the organic solvent listed above, this result implied that 3-NPA or 3-NPOH exists in the plant mainly as conjugated forms, water or acid in the solvent could release them. This was proved by sequential extraction with water or acid followed by organic solvents. As Table 1 showed the amount determined in the same sample was significantly higher in the sequential extraction, suggesting pure water or acid is more efficient in releasing 3-NPA from the conjugates, though only a trace amount of 3-NPOH was found in all the extracts. In literature, inorganic acid (from 0.6 mol/L HCl to 2 mol/L of H_2_SO_4_) had been used to hydrolysis the conjugates of 3-NPA and 3-NPOH in species of *Astragalus* from North American and European herbaria [5,7,11]. There have been reports that enzymes could release 3-NPOH from its conjugate forms in literature. For example, the microbial β-glucosidase and esterase in the rumen could rapidly hydrolyze and liberate free 3-NPA and 3-NPOH from their conjugates in the in vivo experiment [12]. 3-NPOH was found to be released very quickly from miserotoxin when mixed with rumen fluid in in vitro experiments [13]. One report believed that enzymatic hydrolysis of glycosides of 3-NPOH from miserotoxin was probably the reason why its yield was extremely low while using aqueous extraction [5]. Our experiment indicated that endogenous enzymes in *A. canadensis* could also release 3-NPA or 3-NPOH from the conjugates. Based on this, we hypothesized that by adding water to the freeze dried sample the autohydrolysis process by the enzymes in the plant material itself was induced. This observation led us to experiment with pure water extraction for the determination of 3-NPA and 3-NPOH in freeze dried Canadian milkvetch samples.

**Figure 1:**
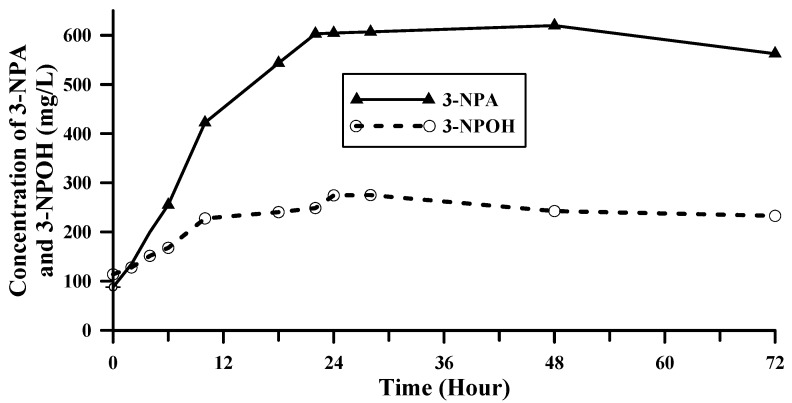
Time course of concentration of 3-NPA and 3-NPOH in the water extract of Canadian milkvetch sample.

Time course of the concentration of 3-NPA and 3-NPOH extracted by water from Canadian milkvetch is shown in Figure 1. It showed that after water was added to the sample, the concentration of 3-NPA and 3-NPOH in the extract kept increasing during the first 24 h and then it reached a plateau. Possible reasons for this could be that time is needed to release 3-NPA or 3-NPOH from other forms of the compounds in the plant material and/or it is a slow distribution equilibrium (24 h) of 3-NPA or 3-NPOH between extraction solvents and the plant material. To determine the most possible reason for this, we compared hot water extraction and room temperature extraction.

Table 2 shows that heated water only extracted 22% of 3-NPA and 65% of 3-NPOH from the Canadian milkvetch samples compared to the water at room temperature, and longer extraction time did not make any difference. More than three times the amount of 3-NPA was extracted by water at room temperature than heated water with a 4 h extraction. This indicated the time course results of the room temperature water extraction were not due to a slow equilibrium, but rather slow release of free 3-NPA and 3-NPOH from the plant material. Since hot water resulted in a significant reduction of the amount of the extracted compounds, it suggested that the release of 3-NPA or 3-NPOH was done by autolysis of the conjugated forms by certain enzyme existing in the plant itself and time is needed for the enzymatic hydrolysis. Heated water stopped the activity of enzymes, extending extraction time would not increase the concentration of 3-NPA or 3-NPOH, the measured 3-NPA or 3-NPOH from heated water most probably came from some non-denatured enzyme activity during the temperature development in the sample. The extractions of Canadian milkvetch samples by pure organic solvents (ethanol acetone and acetonitrile) or 80% organic solvents were not efficient, since the high amount of organic solvents denatured the enzyme. 

As shown in Table 1 the amount of 3-NPA released by 1 mol/L HCl was significantly smaller than that of water extraction, possibly due to the esterification of 3-NPA and 3-NPOH under acidic condition. The detected amount of 3-NPA by sequential extraction of 1 mol/L HCl followed by acetone or acetonitrile showed no significant difference with the water extraction. Besides the more complicated sample preparation by sequential extraction, the peak shape, especially that of 3-NPOH, showed severe band broadening when the sequential extract was injected into HPLC under the developed HPLC condition, as discussed in Section 2.2. All the results supported that water extraction at room temperature is the best procedure for the analysis of 3-NPA and 3-NPOH in freeze dried Canadian milkvetch samples. Our experiment supports that water extraction at room temperature for 24 h is the optimal procedure for extraction of 3-NPA and 3-NPOH from freeze dried Canadian milkvetch samples. Therefore, this procedure was adopted for batch sample treatment for 3-NPA and 3-NPOH analysis in freeze dried Canadian milkvetch. However, one special note for this extraction procedure is that it could only be applied to fresh or freeze dried plant materials as it depends on the endogenous enzymes. Extended storage or drying at elevated temperature could lead to deactivation of the enzymes, and therefore the proposed water extraction will not lead to the total release of free 3-NPA or 3-NPOH. 

### 2.2. HPLC Conditions for the Analysis of 3-NPA and 3-NPOH

**Figure 2:**
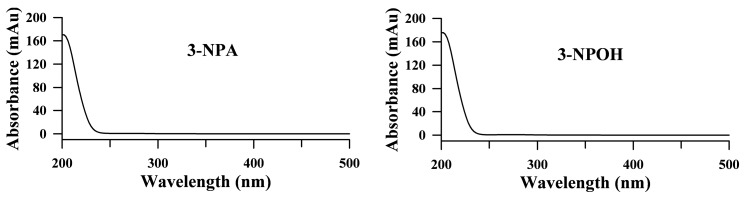
UV spectra of 3-nitropropanoic acid (3-NPA) and 3-nitropropanol (3-NPOH).

Both 3-NPA and 3-NPOH are polar small molecules and hardly retained on the reverse phase, such as the most commonly used C18 columns. Here we chose a Phenomenex Kinetex 2.6 μ F5 100 Å 100 × 4.6 mm column to study the retention and separation of 3-NPA and 3-NPOH. Combining core-shell technology and five interaction mechanisms with Pentafluorophenyl with TMS endcapping as stationary phase, this column has been proven for polar compound retention with polar mobile phase. The UV spectra of 3-NPA and 3-NPOH (Figure 2) showed that the optimal UV absorption of 3-NPA and 3-NPOH is 210 nm and at higher wavelength, the absorbance dramatically dropped. Therefore a very low detection wavelength (e.g., 210 nm) was chosen to measure both compounds. This has limited the choice of mobile phase and modifiers too. UV transparent solvents like water and acetonitrile are preferred to methanol, acetone, etc. and isocratic elution is preferred to gradient elution. 

**Figure 3:**
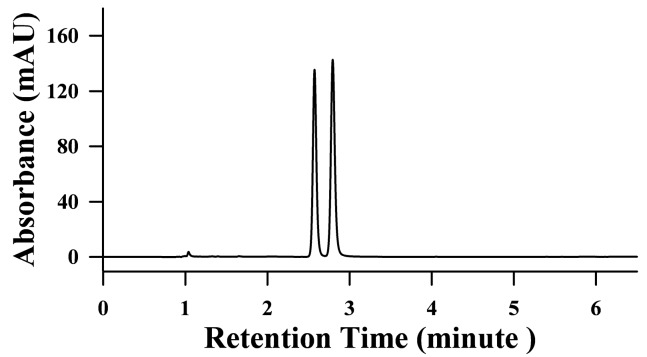
A typical HPLC chromatogram of mixture standards of 3-NPA and 3-NPOH at 20 mg/L in water with pH 3.0 12.5 mM phosphoric buffer as mobile phase on a Phenomenex Kinetex 2.6 μ F5 100 Å column.

Due to the acidic nature of the compounds, acidic buffers have been tried for the separation of the two compounds. With mobile phase of 10 mM ammonium acetate (pH 6.58) or 10 mM ammonium acetate with 0.02% acetic acid (pH 4.85), 3-NPOH was retained, but 3-NPA was not. When 0.1% acetic acid (pH 2.93) was used as mobile phase, both 3-NPA and 3-NPOH were retained and they had baseline separation, resolution between two peaks was 2.74, the capacity factor of 3-NPA and 3-MPOH were 1.729 and 1.781, respectively. However, a negative peak was present in the chromatograph with this mobile phase at this wavelength, which made it impractical to analyze real samples. Additionally, it only worked for the 3-NPA and 3-NPOH in water or in 0.1% acetic acid, whereas huge solvent peaks appeared and disturbed the separation when 10% acetonitrile, acetone, or ethanol was used as reconstitution solvent. With 25 mM potassium dihydrogen phosphate in water (pH 4.32) used as mobile phase, 3-NPOH were retained, but 3-NPA was still not reasonably retained and the peak of 3-NPOH showed front tailing and peak broadening. However, when 25 mM potassium dihydrogen phosphate in water was adjusted to pH 3.0 with phosphoric acid and used as mobile phase, all the problems were solved. Further studies showed that the blockage of the flow path of 1290 HPLC system occurred sometimes by the salt if not washed immediately with water after use. To solve this problem, potassium dihydrogen phosphate was replaced by ammonium dihydrogen phosphate, and the concentration was halved to 12.5 mM. A typical chromatogram of the standard solution of 20 mg/L 3-NPA and 3-NPOH is shown in Figure 3 with pH 3.0 12.5 mM ammonium phosphoric buffer on a Phenomenex Kinetex 2.6 μ F5 100 Å 100 × 4.6 mm at 210 nm. 

**Figure 4:**
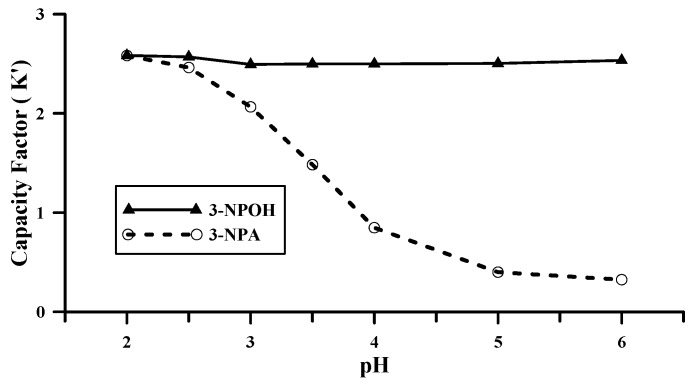
Effect of pH on the capacity factor of 3-NPA and 3-NPOH separated on a Phenomenex Kinetex 2.6 μ F5 100 Å 100 × 4.6 mm column with ammonium phosphate buffer.

The capacity factor (*k*’) of 3-NPA and 3-NPOH were 2.06 and 2.49, respectively with pH 3.0 ammonium phosphate buffer as mobile phase. However, as water extract of a freeze-dried sample is a very complex solution, we need to verify if this condition is specific for the analysis of the plant extracts. This was done through altering the pH of the mobile phase gradually (the pH of the sample was adjusted accordingly) and comparing the UV spectra of the peaks of authentic 3-NPA and 3-NPOH and that of the samples. The chromatograms and the UV spectra of the peaks of the standards, the samples and the spiked samples with elution at different pH were presented in the supplement (Figures S1–S17). Figure S1 showed that the peaks of 3-NPA and 3-NPOH were not separated at pH 2.0, and the *k*’ of 3-NPA and 3-NPOH was the same 2.58. At pH 2.5 the two compounds were not baseline separated (Figure S2) and the *k*’of 3-NPA and 3-NPOH was 2.46 and 2.56 respectively. At pH 3.0, the two peaks of the standards were baseline separated with *k*’of 3-NPA and 3-NPOH 2.06 and 2.49 respectively (Figure S3). At this pH, both of the peaks of 3-NPA and 3-NPOH in the sample were symmetric, the UV spectra of 3-NPA of the standard and the sample were exactly the same (Figure S4), but the spectrum of the peak of 3-NPOH of the sample was not exactly the same as that of the standard (Figure S5), indicating some kind of interference for the analysis of 3-NPOH in the sample under this pH. When the pH was adjusted to 3.5, *k*’ of 3-NPA and 3-NPOH were 1.48 and 2.50 respectively with peaks baseline separated (Figure S6). Both of the UV spectra of 3-NPA and 3-NPOH of the peaks of the sample were exactly the same as that of the standards (Figures S7 and S8), implying the specificity for the analysis of the two compounds under this condition. The side peak eluted just behind the peak of 3-NPOH was possibly the compound overlapped with 3-NPOH at pH 3.0. At pH 4.0, the *k*’ of 3-NPA and 3-NPOH were 0.85 and 2.50 respectively with peaks baseline separated (Figure S9), and the UV spectra of 3-NPA and 3-NPOH of the sample were the same as that of the standard (Figures S10 and S11). At pH 5.0 and 6.0 3-NPA and 3-NPOH were baseline separated (Figures S12 and S15), and the *k*’ of 3-NPA and 3-NPOH were 0.40 and 2.50 respectively at pH 5.0 and 0.32 and 2.53 respectively at pH.6.0. The UV spectra of the peaks of 3-NPA and 3-NPOH of the sample were the same as that of the standards (Figures S13, S14, S16 and S17) at those two pH conditions. However, there was peak tailing under those two pH conditions for 3-NPA and it was also very close to void volume (Figures S12 and S15). The correlation of *k*’ and pH was shown as Figure 4. Taking into the consideration of the above result, pH 3.5–4.0 was demonstrated the optimal pH value for the analysis of 3-NPA and 3-NPOH in real plant samples.

### 2.3. Method Validation

The linearity and LODs and LOQs of the HPLC analysis was done using 3-NPA and 3-NPOH standard solutions, due to the fact that a plant matrix that does not contain 3-NPA and 3-NPOH was not available. Therefore, the linearity and LODs and LOQs of this study are the linearity and sensitivity of the HPLC analysis, and could only be used as reference for real plant samples. The developed HPLC method demonstrated an excellent linear relationship between the peak area and the concentration for both 3-NPA and 3-NPOH with correlation coefficients (*R*^2^) ≥ 0.9997 (Table 3). The linearity ranges covered three orders from 1 to 600 mg/L for both compounds, the LODs were ≤0.038 ng/mL and the LOQs were ≤0.123 ng/mL, which is satisfactory for actual samples analysis. The recovery of each of the compounds in the freeze dried samples was studied by adding a certain amount of 3-NPA and 3-NPOH water stock solution to the freeze dried samples and the spiked samples went through the analytical procedure including sample preparation and HPLC analysis as developed in this study. The spiking level included 100, 400, and 1000 ng/mg dry weight (DW). The results are listed in Table 4. Both compounds at the different spiking levels showed good recoveries, ranging from 97.4 to 116.8%, indicating the accuracy of the method. The obtained RSD values of the procedure were lower than 3.28% for both compounds in the real samples, indicating the high precision of the method (Table 5). These data indicate that the method is highly accurate and reproducible. Moreover, the developed method is much simpler and has a lower cost when compared to the traditionally used spectrometric method or TLC method. The extract compounds can be baseline separated on the Phenomenex Kinetex 2.6 μ F5 100 Å 100 × 4.6 mm column in 6 min. All these suggest that the method is valid and highly efficient, therefore can be used to analyze 3-NPA and 3-NPOH in freeze dried Canadian milkvetch. 

## 3. Conclusions

In conclusion, a simple and efficient procedure for the determination of 3-nitropropanoic acid and 3-nitropropanol in freeze dried Canadian milkvetch has been developed through this study. Since those two compounds exist as both aglycones and conjugated forms, water extraction for 24 h with shaking has been proven to be sufficient to release and extract them from the freeze-dried plant material. The extracted compounds could be based line separated without any interference on a Phenomenex Kinetex 2.6 μ F5 100 Å 100 × 4.6 mm column with pH 3.5 phosphonate buffer as mobile phase, and can be detected at 210 nm. The developed method has been validated for its accuracy, repeatability, and robustness. It has been successfully applied for batch sample analysis. 

## 4. Materials and Methods 

### 4.1. Chemicals and Reagents

3-nitro-1-propanoic acid was purchased from Sigma-Aldrich (St. Louis, MO, USA), and 3-nitro-1-propanol was from Merck Millipore (Darmstadt, Germany). Stock solutions of each standard were prepared in methanol or water at 2.0 mg/mL level and were stored at −20 °C. Stock solution of mixed standards was prepared by mixing each individual standard stock solution in equal volume with a final concentration at 1.0 mg/L level. HPLC grade acetonitrile, methanol, and dichloromethane were supplied by Caledon Laboratories Chemicals (Georgetown, DC, Canada). High purity water was made by a Barnstead Nanopure purification system (Thermo Scientific, Dubuque, IA, USA). *A. canadensis* was harvested from the experimental field of the Swift Current Research and Development Centre of Agriculutre-Agri-Food Canada in Saskatchewan, SK, Canada. The sample was then freeze dried and sheared with a SmartGrind (Black & Decker Inc., Mississauga, ON, USA) for about 2 min, sieved through an 80 mesh sieve, and then stored at −20 °C for further analysis. 

### 4.2. Extraction of 3-NPA and 3-NPOH from A. canadensis 

Extraction procedures used in this study include one-step solvent extraction, two-step sequential extraction and pure water extraction. The solvent systems used in one-step extraction include (Table 1), 100% acetone, 100% acetonitrile, 100% ethanol, mixture of water and acetone (2:8), mixture of water and acetonitrile (2:8), mixture of water and ethanol (2:8), 1 mol/L HCl and acetone (2:8), 1 mol/L HCl and acetonitrile (2:8), and 1 mol/L HCl and ethanol (2:8). The procedure of one step extraction is that 0.5 gram of dried *A. canadensis* was vortexed with 10 mL each of the above solvent system at room temperature for 1 min and then shaken for 4 h at Heidolph Titramax 1000 platform shaker (Heidolph North America, Elk Grove Village, IL, USA). The suspension was centrifuged at 3000 g for 15 min at room temperature, the supernatant was then filtered through a 0.45 μm PVDF filter (Whatman, Sanford, ME, USA) and stored at 4° before HPLC analysis or further processing within one week.

The procedure for two-step sequential extraction is that, to 0.50 gram of freeze dried *A. canadensis*, a certain amount of first solvent was added and vortexed for one minute and the mixture was shaken at room temperature for 4.0 h and then the second solvent was added. The second extraction was also conducted on a Heidolph Titramax 1000 platform shaker (Heidolph North America, Elk Grove Village, IL, USA) at room temperature for 2.0 h. After centrifugation at 3000 *g* for 15 min at room temperature, the supernatant was collected and filtered through a 0.45 μm PVDF filter (Whatman, Sanford, ME, USA) and stored at 4 °C before HPLC analysis or further processing within one week. The solvent system used in the two step sequential extraction includes (Table 1): 2 mL water followed by 8 mL acetone, 2 mL of water followed by 8 mL acetonitrile, 2 mL of water followed by 8 mL ethanol, 2 mL of 1 mol/L HCl followed by 8 mL acetone, 2 mL of 1 mol/L HCl followed by 8 mL acetonitrile, 2 mL of 1 mol/L HCl followed by 8 mL ethanol.

Water extract: To 0.50 g dried *A. canadensis* sample in a 15 mL centrifuge tube 10.0 mL Milli-Q water was added. The mixture was then vortexed and kept on shaking on a Heidolph MultiReax (Heidolph North America, Elk Grove Village, IL, USA) at a speed of 1500 rpm at room temperature. At a different time, a 200 μL of sample was quickly pipetted from the middle of the centrifuge tube into a Ultrafree-MC microcentrifuge filter (Sigma-Aldrich, Mississauga, ON, Canada, Capacity 500 μL durapore PVDF membrane, pore size 0.22 μm), the microcentrifuge filter was centrifuged at 11,000 *g* at an Eppendorf centrifuge 5417R (Eppendorf Canada, Mississauga, ON, Canada) with temperature set at 4 °C for 10 min. The filtrate was then diluted 10 times and transferred to an autosampler vial with inserts for HPLC analysis.

Hot water extract: To evaluate the effect of heated water on the extraction of 3-NPA and 3-NPOH from Canadian milkvetch, 10 mL of boiling water was added to 0.50 g of dry Canadian milkvetch samples in a Supelco 15 mL of clear glass vial, 10.0 mL of boiling water was added and capped with PTFE/Silicone lined cap, the mixture were then put into a heating block (Boekel Industries Inc. Feasterville-Trevose, PA, USA,), and the temperature was kept at 95 °C. The mixture was manually shaken frequently. After pre-set time, the mixture were removed from the block, cooled down and centrifuged at 3000 rpm for 15 min, each supernatant was diluted 10 times and transferred to an HPLC autosampler vial and concentration of 3-NPA and 3-NPOH was determined by HPLC. To compare, water at room temperature was used to extract Canadian milkvetch.

### 4.3. HPLC Conditions

HPLC conditions: An Agilent 1290 series HPLC (Agilent Technology, Palo Alto, CA, USA) equipped with a binary pump, an inline degasser, a thermostatic autosampler, and a DAD was used for the identification and quantification of 3-NPA and 3-NPOH in the samples. A Phenomenex Kinetex 2.6 μ F5 100 Å 100 × 4.6 mm column (Torrance, CA, USA) was used for the separation of 3-NPA and 3-NPOH. The isocratic mobile phase was ammonium phosphate buffer at different pH at a flow rate of 1.0 mL/min for a run time of 6.5 min. The injection volume was 1 or 2 μL for all standards and samples. The DAD collected data from 190–600 nm, and absorbance at 210 nm only was used to monitor and quantify 3-NPA and 3-NPOH. 3-NPA and 3-NPOH were identified by a combination of the retention time in HPLC chromatograms, and UV spectra pattern of pure standard compounds.

### 4.4. Quantification

All calibration solutions (1.00, 2.00, 5.0, 10.0, 20.0, 50.0, 100.0, and 200.0 mg/L) were prepared by serial dilution of the individual stock solutions with water. 2 μL of each solution were injected into the HPLC system, all in duplicate, and a calibration curve was generated between the HPLC peak area of the compound and the concentration. Peak areas were integrated automatically and the correlation coefficients were obtained by ChemStation^®^ (Rev. C. 01.05 [35], Agilent Technology, Palo Alto, CA, USA). The contents of 3-NPA and 3-NPOH were expressed in mg/g DW.

### 4.5. Method Validation

The instrumental linearity was determined with known concentrations of mixed standard solutions (1.00, 2.00, 5.0, 10.0, 20.0, 50.0, 100.0, 200.0, 500.0, and 600.0 mg/L). 1 μL of each solution were directly injected into the HPLC system, all in triplicate, and a correlation was generated between the HPLC peak area of the compound and the concentration. The precision of the method was evaluated by analyzing the same sample (*n* = 6) six times and the concentration of each compound in the sample was determined and the relative standard deviations were calculated. The recovery rate was conducted according to the standard addition method [14]. Water stock solutions of the standards were added to the freeze dried *A. canadensis* samples at different concentrations. The spiked samples went through the sample preparation and HPLC analysis and the recovery percentage was determined. The recovery experiment was done in triplicate. To determine the limit of detection (LOD) and limit of quantification (LOQ) of the HPLC analysis, low concentrations of working standard solutions of 3-NPA and 3-NPOH were prepared by series dilution of 1 mg/mL of stock solution. Six replicates of 2 μL of working standards and water blank were injected into the HPLC. Peak area and peak height were recorded. Limit of quantitation (LOQ) were calculated with 10 times the signal to noise of peak height and limit of detection LOD with 3 times the signal to noise of peak height. 

## Figures and Tables

**Table 1:** Measured amount of 3-NPA (mg/g) in freeze dried Canadian milkvetch sample by different extraction.

Extract	H_2_O:Acetone (2:8)	H_2_O:Acetonitrile (2:8)	H_2_O:Ethanol (2:8)	1 mol/L HCL:Acetone (2:8)	1 mol/L HCL:Acetonitrile (2:8)	1 mol/L HCL:Ethanol (2:8)	1 mol/L HCL	H_2_O
One-Time Extraction	1.4	2.3	2.3	2.9	3.2	2.0	4.6	9.3
Sequential Extraction	6.2	6.8	6.2	9.8	10.3	4.2		

**Table 2:** Effect of water temperature on the measured content of 3-NPA and 3-NPOH (mg/g) in freeze dried Canadian milkvetch sample.

Temperature	Time (H)	3-NPA	3-NPOH
95 °C	1.5	2.02	0.15
95 °C	4	2.49	0.15
95 °C	48	2.31	0.14
25 °C	4	8.48	0.20
25 °C	48	9.27	0.23

**Table 3:** Data of method validation of the proposed method for the analysis of 3-NPA and 3-NPOH in Canadian milkvetch ^1^.

Compounds	Linear Range (μg/mL)	*R*^2^	LOD (μg/mL)	LOQ(μg/mL)
3-NPA	1–600	0.9997	0.038	0.123
3-NPOH	1–600	0.9998	0.036	0.116

^1^ *R*^2^: determination coefficient; LOD: limit of detection; LOQ: limit of quantification.

**Table 4:** Recoveries of 3-NPA and 3-NPOH following the developed procedure by standard addition method (*n* = 3).

Compounds	Percent Recoveries (%) in in Freeze Dried Canadian Milkvetch Samples at Different Spiking Levels (ng/mg DW)		
	100	400	1000
3-NPA	100.09	102.7	106.8
3-NPOH	97.4	116.8	103.5

**Table 5:** Relative standard deviation (%) of the concentration of 3-NPA and 3-NPOH in the Canadian milkvetch sample analyzed by the proposed procedure (*n* = 5).

Compounds	Measured Amount (mg/g)	RSD (%)
3-NPA	16.65	1.91
3-NPOH	1.20	3.28

## Supplementary Materials

The following are available online at www.mdpi.com/2072-6651/9/7/204/s1, Figure S1: Overlaid HPLC chromatograms of mixture standards of 3-NPA and 3-NPOH at 10 ppm in water and spiked Canadian milkvetch sample, with pH 2.0 12.5 mM ammonium phosphate buffer as moble phase on a Phenomenex Kinetex 2.6 µ F5 100 Å column, Figure S2: Overlaid HPLC chromatograms of mixture standards of 3-NPA and 3-NPOH at 10 ppm in water and spiked Canadian milkvetch sample, with pH 2.5 12.5 mM ammonium phosphate buffer as moble phase on a Phenomenex Kinetex 2.6 µ F5 100 Å column, Figure S3: Overlaid HPLC chromatograms of mixture standards of 3-NPA and 3-NPOH at 10 ppm in water, Canadian milkvetch sample and spiked Canadian milkvetch samples, with pH 3.0 12.5 mM ammonium phosphate buffer as moble phase on a Phenomenex Kinetex 2.6 µ F5 100 Å column, Figure S4: UV spectra of the peak of 3-NPA in Canadian milkvetch under the HPLC condition with pH 3.0 12.5 mM ammonium phosphate buffer as moble phase on a Phenomenex Kinetex 2.6 µ F5 100 Å column, Figure S5: UV spectra of the peak of 3-NPOH in Canadian milkvetch under the HPLC condition with pH 3.0 12.5 mM ammonium phosphate buffer as moble phase on a Phenomenex Kinetex 2.6 µ F5 100 Å column, Figure S6: Overlaid HPLC chromatograms of mixture standards of 3-NPA and 3-NPOH at 10 ppm in water, Canadian milkvetch sample and spiked Canadian milkvetch samples, with pH 3.5 12.5 mM ammonium phosphate buffer as moble phase on a Phenomenex Kinetex 2.6 µ F5 100 Å column, Figure S7: UV spectra of the peak of 3-NPA in Canadian milkvetch under the HPLC condition with pH 3.5 12.5 mM ammonium phosphate buffer as moble phase on a Phenomenex Kinetex 2.6 µ F5 100 Å column, Figure S8: UV spectra of the peak of 3-NPOH in Canadian milkvetch under the HPLC condition with pH 3.5 12.5 mM ammonium phosphate buffer as moble phase on a Phenomenex Kinetex 2.6 µ F5 100 Å column, Figure S9: Overlaid HPLC chromatograms of mixture standards of 3-NPA and 3-NPOH at 10 ppm in water, Canadian milkvetch sample and spiked Canadian milkvetch samples, with pH 4.0 12.5 mM ammonium phosphate buffer as moble phase on a Phenomenex Kinetex 2.6 µ F5 100 Å column, Figure S10: UV spectra of the peak of 3-NPA in Canadian milkvetch under the HPLC condition with pH 4.0 12.5 mM ammonium phosphate buffer as moble phase on a Phenomenex Kinetex 2.6 µ F5 100 Å column, Figure S11: UV spectra of the peak of 3-NPOH in Canadian milkvetch under the HPLC condition with pH 4.0 12.5 mM ammonium phosphate buffer as moble phase on a Phenomenex Kinetex 2.6 µ F5 100 Å column, Figure S12: Overlaid HPLC chromatograms of mixture standards of 3-NPA and 3-NPOH at 10 ppm in water, Canadian milkvetch sample and spiked Canadian milkvetch samples, with pH 5.0 12.5 mM ammonium phosphate buffer as moble phase on a Phenomenex Kinetex 2.6 µ F5 100 Å column, Figure S13: UV spectra of the peak of 3-NPA in Canadian milkvetch under the HPLC condition with pH 5.0 12.5 mM ammonium phosphate buffer as moble phase on a Phenomenex Kinetex 2.6 µ F5 100 Å column, Figure S14: UV spectra of the peak of 3-NPOH in Canadian milkvetch under the HPLC condition with pH 5.0 12.5 mM ammonium phosphate buffer as moble phase on a Phenomenex Kinetex 2.6 µ F5 100 Å column, Figure S15: Overlaid HPLC chromatograms of mixture standards of 3-NPA and 3-NPOH at 10 ppm in water, Canadian milkvetch sample and spiked Canadian milkvetch samples, with pH 6.0 12.5 mM ammonium phosphate buffer as moble phase on a Phenomenex Kinetex 2.6 µ F5 100 Å column, Figure S16: UV spectra of the peak of 3-NPA in Canadian milkvetch under the HPLC condition with pH 6.0 12.5 mM ammonium phosphate buffer as moble phase on a Phenomenex Kinetex 2.6 µ F5 100 Å column, Figure S17: UV spectra of the peak of 3-NPOH in Canadian milkvetch under the HPLC condition with pH 6.0 12.5 mM ammonium phosphate buffer as moble phase on a Phenomenex Kinetex 2.6 µ F5 100 Å column.

## References

[1] Williams M.C., James L.F. (1975). Toxicity of nitro-containing astragalus to sheep and chicks. J. Range Manag..

[2] Williams M.C., Stermitz F.R., Thomas R.D. (1975). Nitro compounds in astragalus species. Phytochemistry.

[3] Benn M., Bai Y., Majak W. (1995). Aliphatic nitro-compounds in astragalus canadensis. Phytochemistry.

[4] Majak W., Pass M.A., Cheeke P.R. (1989). Aliphatic nitrocompounds. Toxicants of Plant Origin.

[5] Majak W., Bose R.J. (1974). Chromatographic methods for the isolation of miserotoxin and detection of aliphatic nitro compounds. Phytochemistry.

[6] Matsumoto H., Unrau A.M., Hylin J.W., Temple B. (1961). Spectrophotometric determination of 3-nitropropanoic acid in biological extracts. Anal. Chem..

[7] Stermitz F.R., Lowry W.T., Norris F.A., Buckeridge F.A., William M.C. (1972). Aliphatic nitro compounds from *astragalus species*. Phytochemistry.

[8] Greenwood D.R. (1990). Determination of aliphatic nitro compounds in roots of lotus pedunculatus-the effect of maceration on leves of components. J. Sci. Food Agric..

[9] Gilbert M., Penel A., Kosikowski F.V., Henion J.D., Maylin G.A., Lisk D.J. (1977). Electron affinity gas chromatographic determination of beta nitropropionic acid as its pentafluorobenzyl derivative in cheeses and mold filtrates. J. Food Sci..

[10] Muir A.D., Majak W. (1984). Quantitative determination of 3-nitropropionic acid and 3-nitropropanol in plasma by hplc. Toxicol. Lett..

[11] William M.C. (1982). 3-Nitropropionic acid and 3-nitro-1-propanol in species of *astragalus*. Can. J. Bot..

[12] Anderson R.C., Rasmussen M.A., Allison M.J. (1993). Metabolism of the plant toxins nitropropionic acid and nitropropanol by ruminal microorganisms. Appl. Environ. Microbiol..

[13] Majak W., Clark L.J. (1980). Metabolism of aliphatic nitro compounds in bovine rumen fluid. Can. J. Anim. Sci..

[14] Burns D.T., Danzer K., Townshend A. (2002). Use of the terms “recovery” and “apparent recovery” in analytical procedures (iupac recommendations 2002). Pure Appl. Chem..

