# Supplementary material for: A Simple and Fast Procedure to Determine 3-Nitropropanoic Acid and 3-Nitropropanol in Freeze Dried Canadian Milkvetch (Astragalus canadensis)
Source: Toxins (Basel). 2017 Jun 29;9(7):204. doi: 10.3390/toxins9070204 (PMC5535151; doi:10.3390/toxins9070204)
Supplement: Supplementary file 1 [file toxins-09-00204-s001.pdf]

Supplementary Materials: A Simple and Fast Procedure to Determine 3-Nitropropanoic Acid and 3-Nitropropanol in Freeze Dried Canadian Milkvetch (*Astragalus canadensis*)

Huaizhi Liu, Suqin Shao and Mike Schellenberg

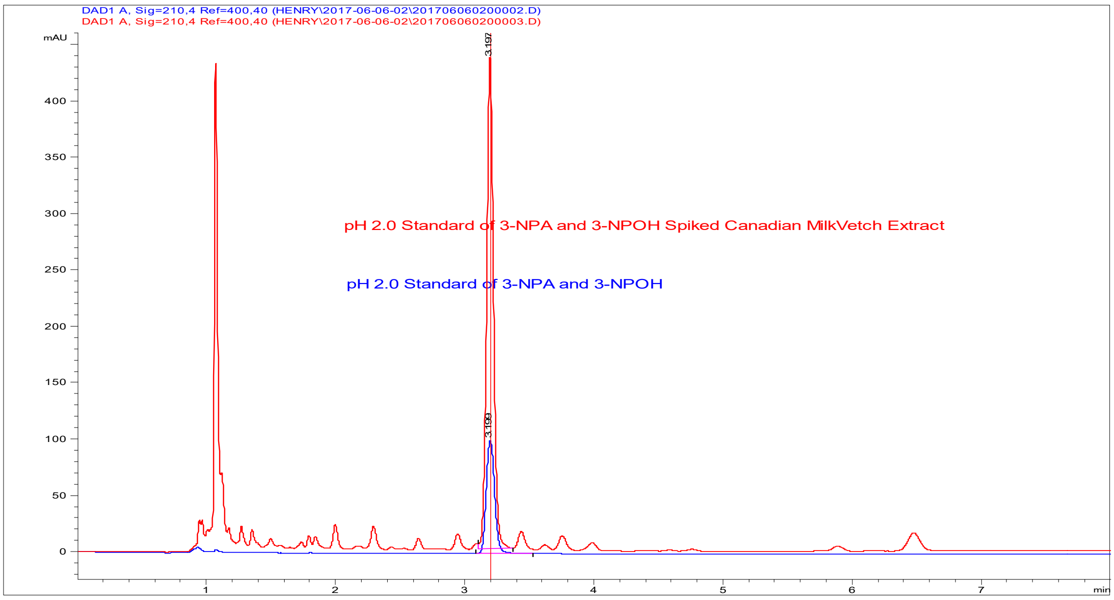

Figure S1. Overlaid HPLC chromatograms of mixture standards of 3-NPA and 3-NPOH at 10 ppm in water and spiked Canadian milkvetch sample, with pH 2.0 12.5 mM ammonium phosphate buffer as moble phase on a Phenomenex Kinetex 2.6 µ F5 100Å column. All standard solution and Canadian milkvetch samples were recon at the corresponding pH mobile phase. 3-NPA and 3-NPOH was not separated.

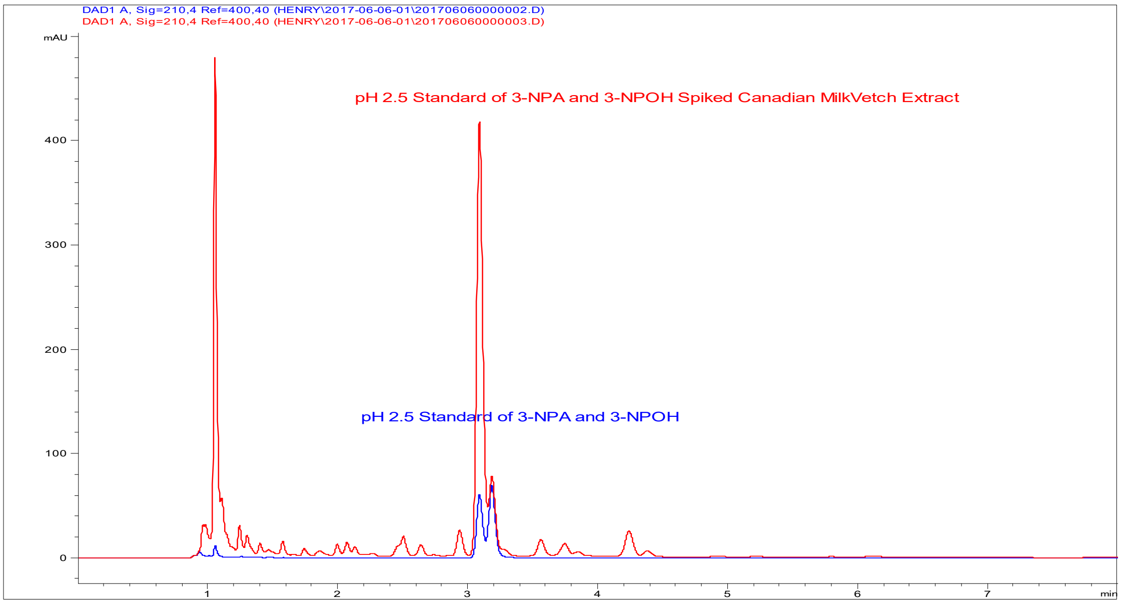

Figure S2. Overlaid HPLC chromatograms of mixture standards of 3-NPA and 3-NPOH at 10 ppm in water and spiked Canadian milkvetch sample, with pH 2.5 12.5 mM ammonium phosphate buffer as moble phase on a Phenomenex Kinetex 2.6 µ F5 100Å column. All standard solution and Canadian milkvetch samples were recon at the corresponding pH mobile phase. 3-NPA and 3-NPOH was not baseline separated.

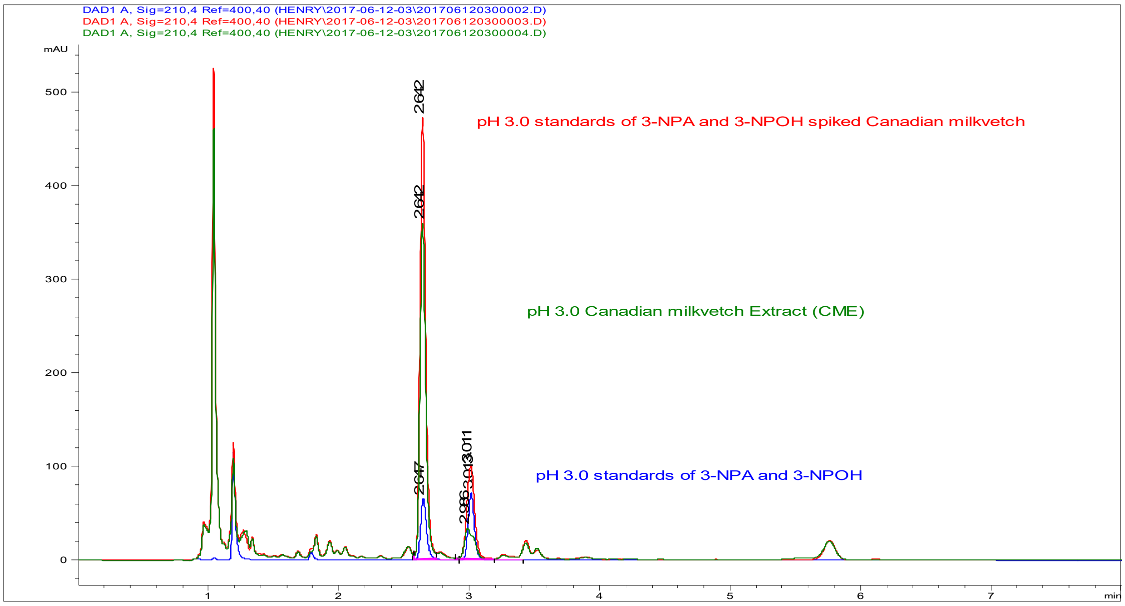

Figure S3. Overlaid HPLC chromatograms of mixture standards of 3-NPA and 3-NPOH at 10 ppm in water, Canadian milkvetch sample and spiked Canadian milkvetch samples, with pH 3.0 12.5 mM ammonium phosphate buffer as moble phase on a Phenomenex Kinetex 2.6 µ F5 100Å column. All standard solution and Canadian milkvetch samples were recon at the corresponding pH mobile phase. 3-NPA and 3-NPOH was baseline separated.

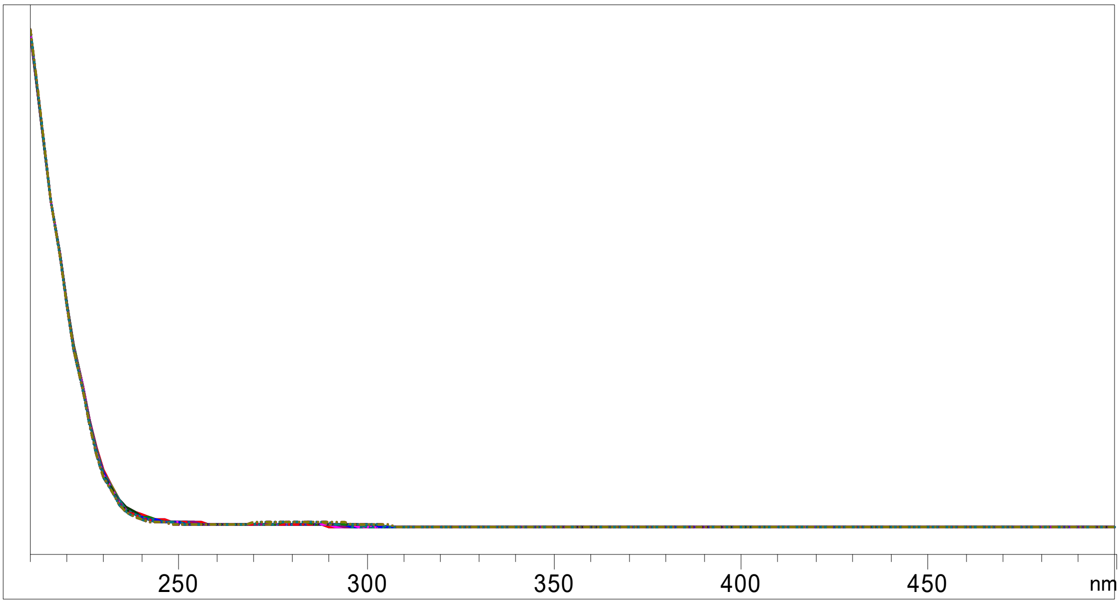

Figure S4. UV spectra of the peak of 3-NPA in Canadian milkvetch under the HPLC condition with pH 3.0 12.5 mM ammonium phosphate buffer as moble phase on a Phenomenex Kinetex 2.6 µ F5 100Å column.

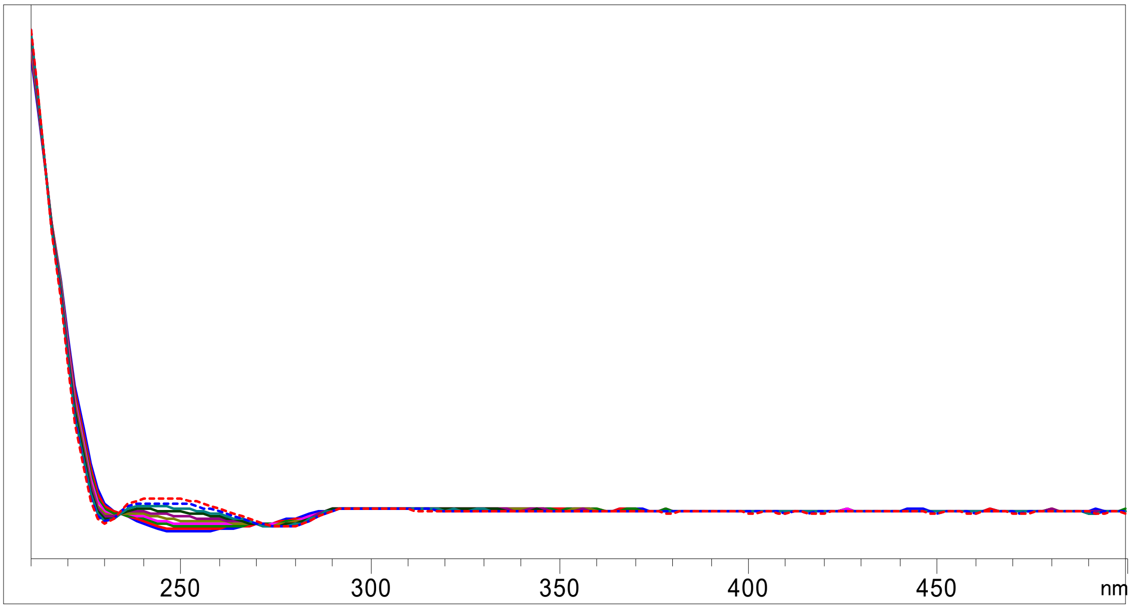

Figure S5. UV spectra of the peak of 3-NPOH in Canadian milkvetch under the HPLC condition with pH 3.0 12.5 mM ammonium phosphate buffer as moble phase on a Phenomenex Kinetex 2.6 µ F5 100Å column.

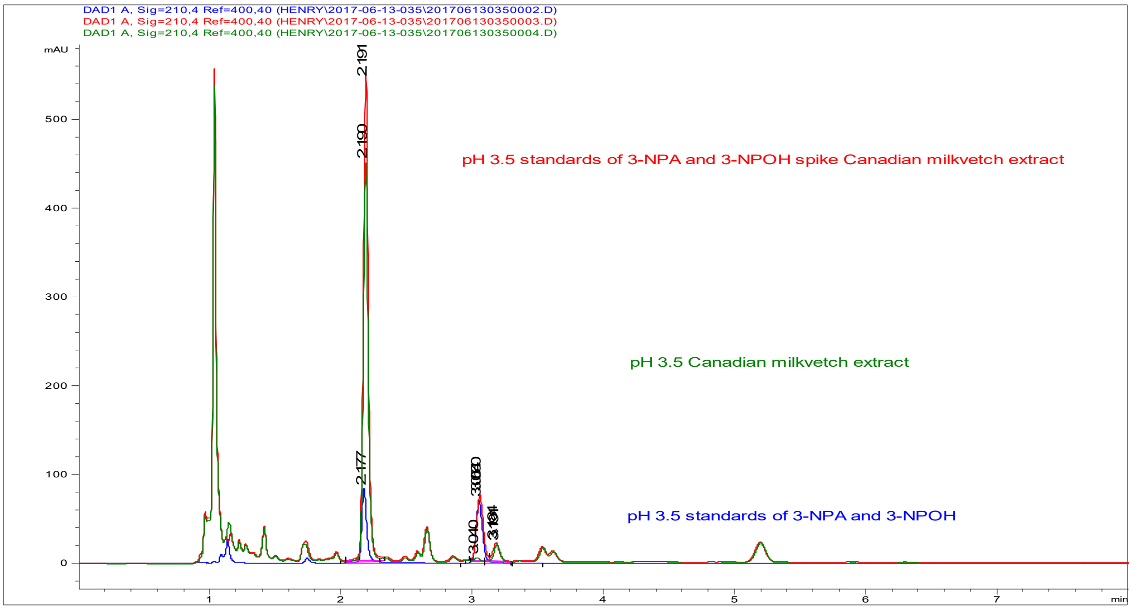

Figure S6. Overlaid HPLC chromatograms of mixture standards of 3-NPA and 3-NPOH at 10 ppm in water, Canadian milkvetch sample and spiked Canadian milkvetch samples, with pH 3.5 12.5 mM ammonium phosphate buffer as moble phase on a Phenomenex Kinetex 2.6 µ F5 100Å column. All standard solution and Canadian milkvetch samples were recon at the corresponding pH mobile phase. 3-NPA and 3-NPOH was baseline separated.

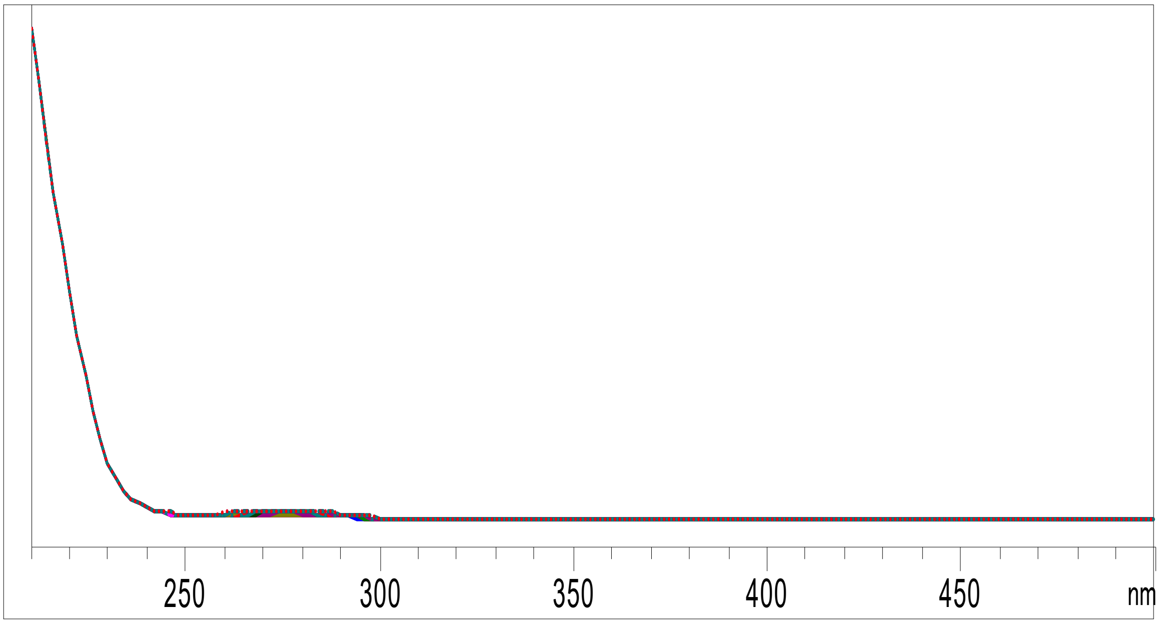

Figure S7. UV spectra of the peak of 3-NPA in Canadian milkvetch under the HPLC condition with pH 3.5 12.5 mM ammonium phosphate buffer as moble phase on a Phenomenex Kinetex 2.6 μ F5 100Å column.

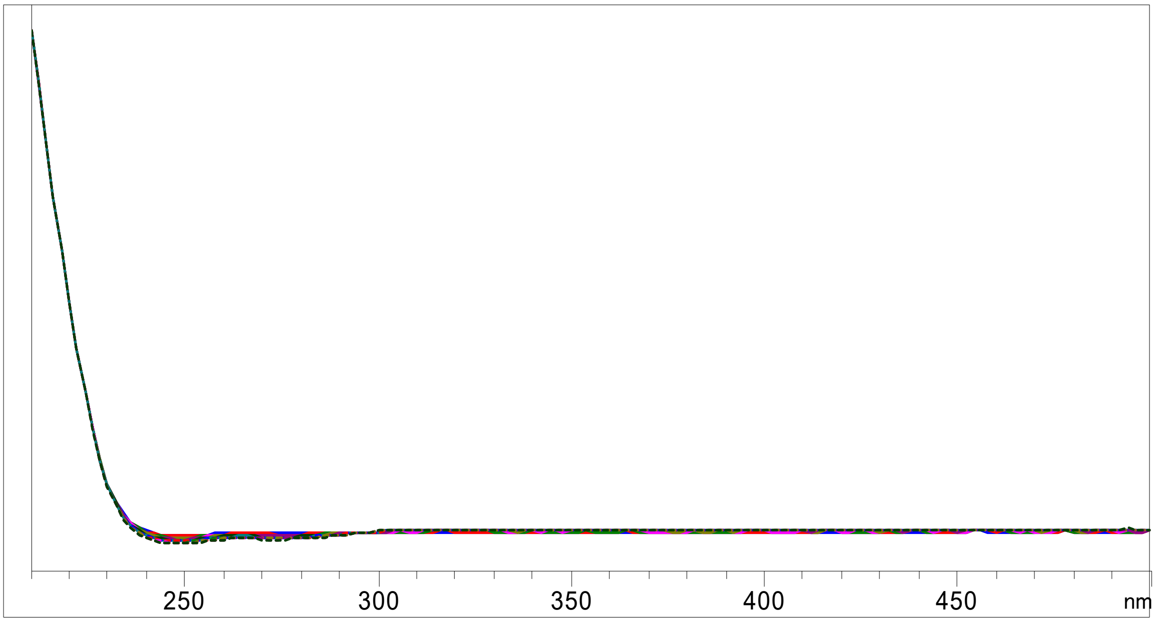

Figure S8. UV spectra of the peak of 3-NPOH in Canadian milkvetch under the HPLC condition with pH 3.5 12.5 mM ammonium phosphate buffer as moble phase on a Phenomenex Kinetex 2.6 μ F5 100Å column.

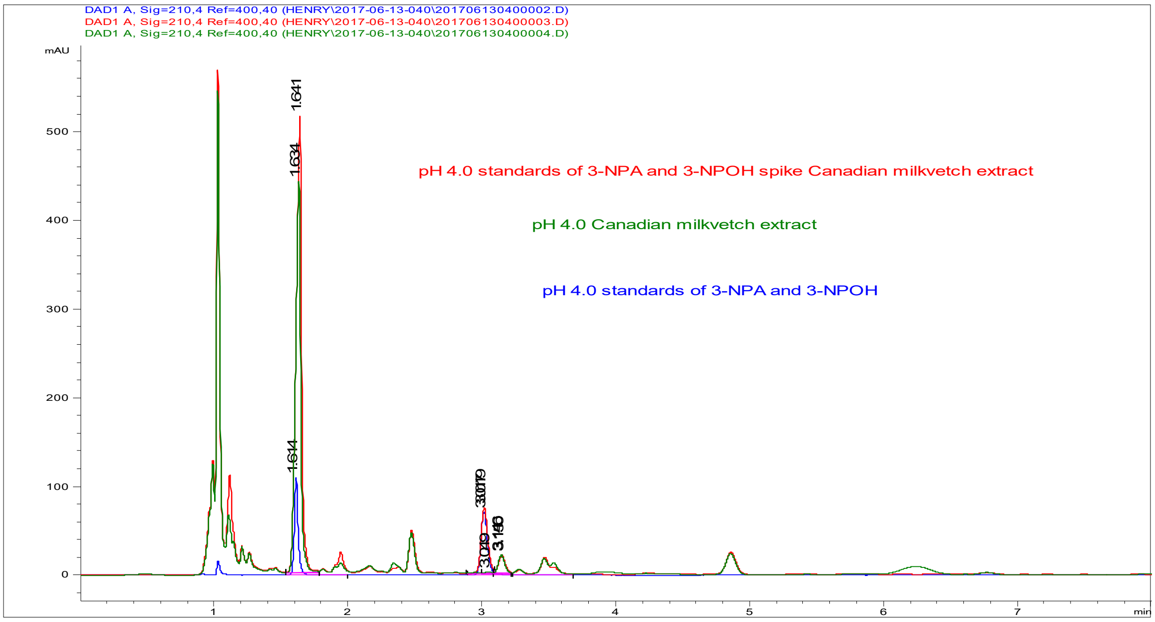

Figure S9. Overlaid HPLC chromatograms of mixture standards of 3-NPA and 3-NPOH at 10 ppm in water, Canadian milkvetch sample and spiked Canadian milkvetch samples, with pH 4.0 12.5 mM ammonium phosphate buffer as moble phase on a Phenomenex Kinetex 2.6 μ F5 100Å column. All standard solution and Canadian milkvetch samples were recon at the corresponding pH mobile phase. 3-NPA and 3-NPOH was baseline separated.

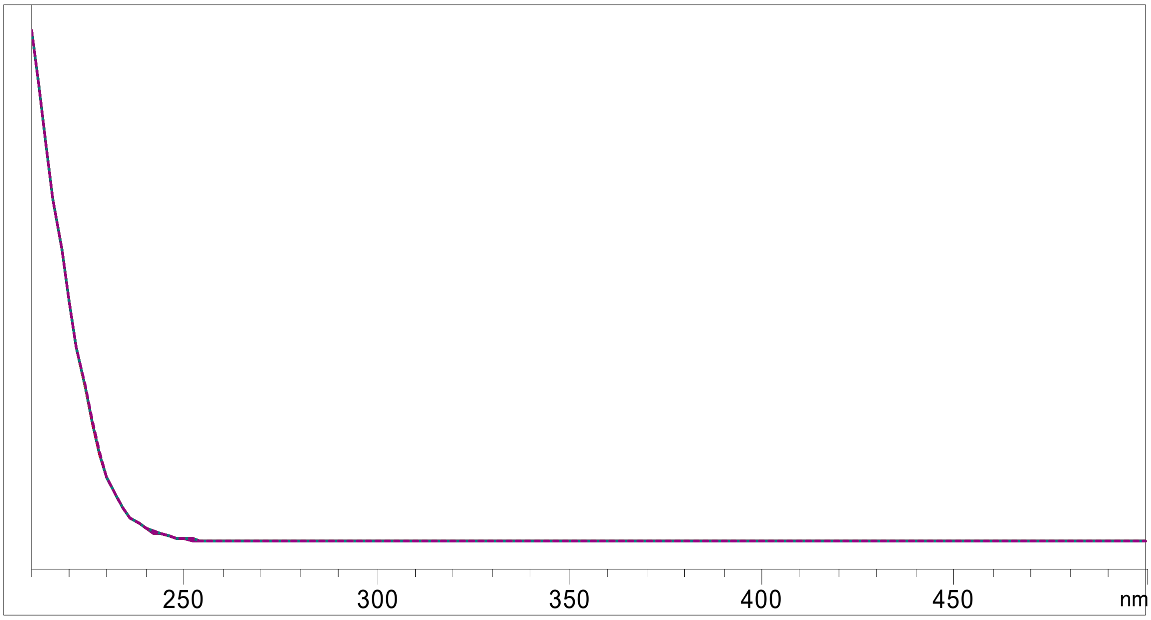

Figure S10. UV spectra of the peak of 3-NPA in Canadian milkvetch under the HPLC condition with pH 4.0 12.5 mM ammonium phosphate buffer as moble phase on a Phenomenex Kinetex 2.6 μ F5 100Å column.

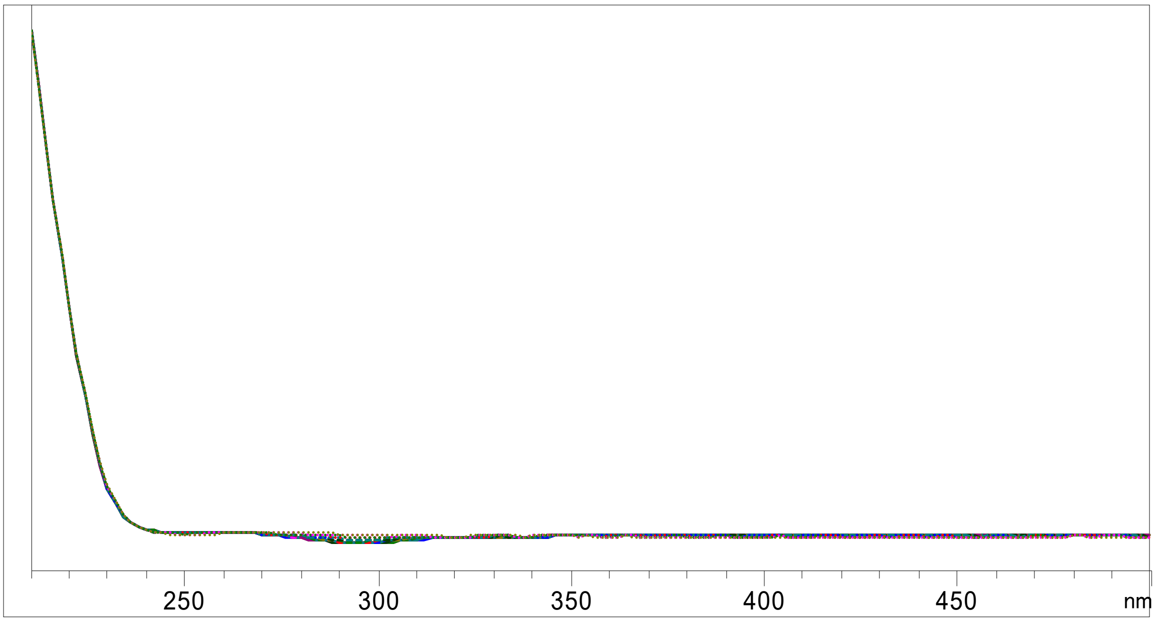

Figure S11. UV spectra of the peak of 3-NPOH in Canadian milkvetch under the HPLC condition with pH 4.0 12.5 mM ammonium phosphate buffer as moble phase on a Phenomenex Kinetex 2.6 µ F5 100Å column.

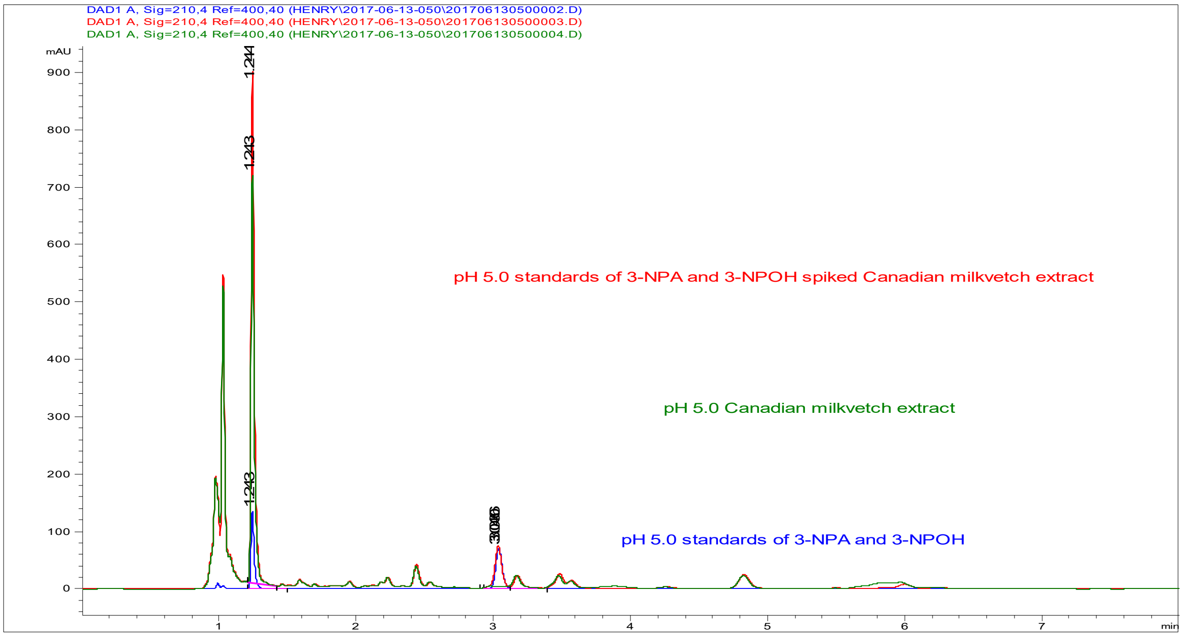

Figure S12. Overlaid HPLC chromatograms of mixture standards of 3-NPA and 3-NPOH at 10 ppm in water, Canadian milkvetch sample and spiked Canadian milkvetch samples, with pH 5.0 12.5 mM ammonium phosphate buffer as moble phase on a Phenomenex Kinetex 2.6 µ F5 100Å column. All standard solution and Canadian milkvetch samples were recon at the corresponding pH mobile phase. 3-NPA and 3-NPOH was baseline separated.

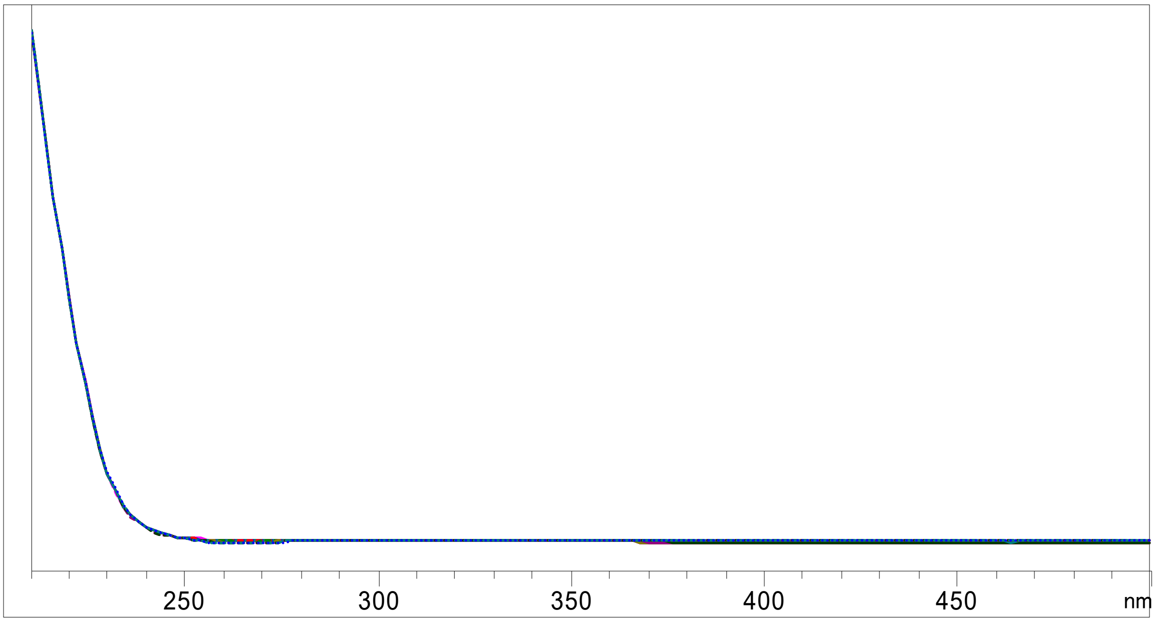

Figure S13. UV spectra of the peak of 3-NPA in Canadian milkvetch under the HPLC condition with pH 5.0 12.5 mM ammonium phosphate buffer as moble phase on a Phenomenex Kinetex 2.6 μ F5 100Å column.

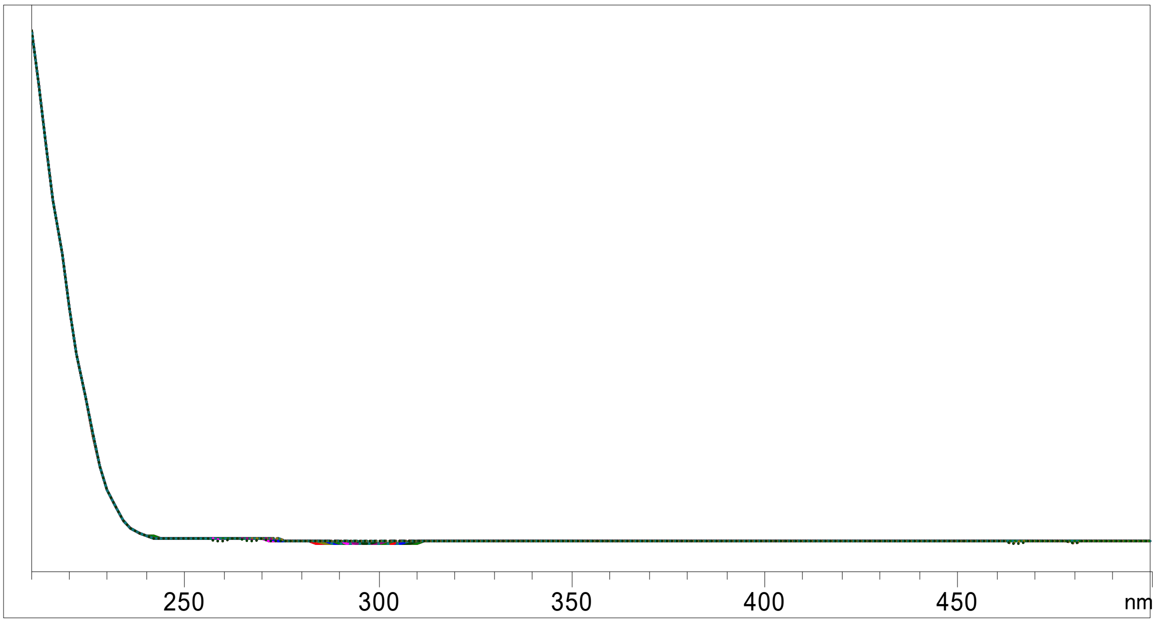

Figure S14. UV spectra of the peak of 3-NPOH in Canadian milkvetch under the HPLC condition with pH 5.0 12.5 mM ammonium phosphate buffer as moble phase on a Phenomenex Kinetex 2.6 μ F5 100Å column.

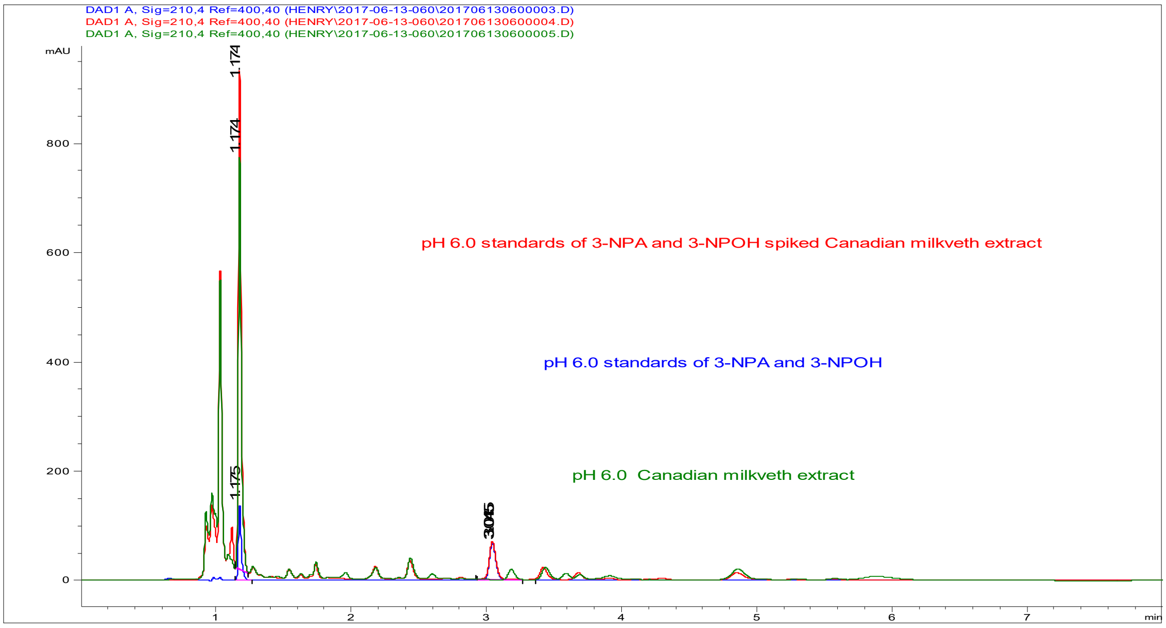

Figure S15. Overlaid HPLC chromatograms of mixture standards of 3-NPA and 3-NPOH at 10 ppm in water, Canadian milkvetch sample and spiked Canadian milkvetch samples, with pH 6.0 12.5 mM ammonium phosphate buffer as moble phase on a Phenomenex Kinetex 2.6 µ F5 100Å column. All standard solution and Canadian milkvetch samples were recon at the corresponding pH mobile phase. 3-NPA and 3-NPOH was baseline separated.

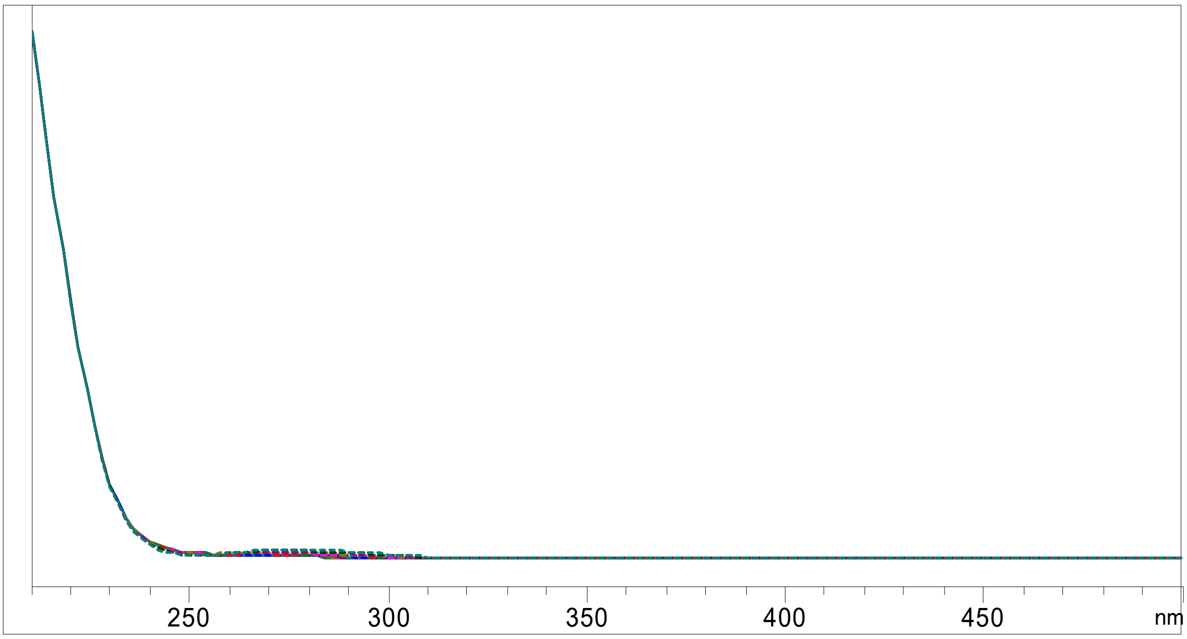

Figure S16. UV spectra of the peak of 3-NPA in Canadian milkvetch under the HPLC condition with pH 6.0 12.5 mM ammonium phosphate buffer as moble phase on a Phenomenex Kinetex 2.6 µ F5 100Å column.

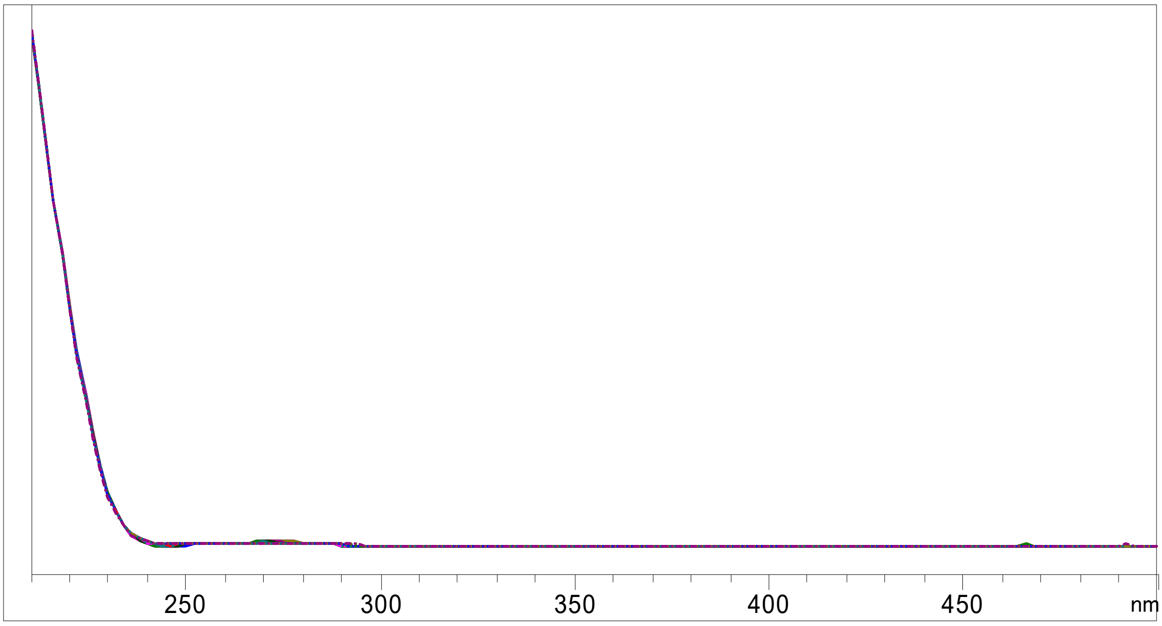

Figure S17. UV spectra of the peak of 3-NPOH in Canadian milkvetch under the HPLC condition with pH 6.0 12.5 mM ammonium phosphate buffer as moble phase on a Phenomenex Kinetex 2.6 µ F5 100Å column.
